# Evolutionary Conservation of Orthoretroviral Long Terminal Repeats (LTRs) and *ab initio* Detection of Single LTRs in Genomic Data

**DOI:** 10.1371/journal.pone.0005179

**Published:** 2009-04-13

**Authors:** Farid Benachenhou, Patric Jern, Merja Oja, Göran Sperber, Vidar Blikstad, Panu Somervuo, Samuel Kaski, Jonas Blomberg

**Affiliations:** 1 Section of Virology, Department of Medical Sciences, Uppsala University, Uppsala, Sweden; 2 Unit of Physiology, Department of Neuroscience, Uppsala University, Uppsala, Sweden; 3 Helsinki Institute for Information Technology, Department of Computer Science, University of Helsinki and Laboratory of Computer and Information Science, Helsinki University of Technology, Helsinki, Finland; University of Hyderabad, India

## Abstract

**Background:**

Retroviral LTRs, paired or single, influence the transcription of both retroviral and non-retroviral genomic sequences. Vertebrate genomes contain many thousand endogenous retroviruses (ERVs) and their LTRs. Single LTRs are difficult to detect from genomic sequences without recourse to repetitiveness or presence in a proviral structure. Understanding of LTR structure increases understanding of LTR function, and of functional genomics. Here we develop models of orthoretroviral LTRs useful for detection in genomes and for structural analysis.

**Principal Findings:**

Although mutated, ERV LTRs are more numerous and diverse than exogenous retroviral (XRV) LTRs. Hidden Markov models (HMMs), and alignments based on them, were created for HML- (human MMTV-like), general-beta-, gamma- and lentiretroviruslike LTRs, plus a general-vertebrate LTR model. Training sets were XRV LTRs and RepBase LTR consensuses. The HML HMM was most sensitive and detected 87% of the HML LTRs in human chromosome 19 at 96% specificity. By combining all HMMs with a low cutoff, for screening, 71% of all LTRs found by RepeatMasker in chromosome 19 were found. HMM consensus sequences had a conserved modular LTR structure. Target site duplications (TG-CA), TATA (occasionally absent), an AATAAA box and a T-rich region were prominent features. Most of the conservation was located in, or adjacent to, R and U5, with evidence for stem loops. Several of the long HML LTRs contained long ORFs inserted after the second A rich module. HMM consensus alignment allowed comparison of functional features like transcriptional start sites (sense and antisense) between XRVs and ERVs.

**Conclusion:**

The modular conserved and redundant orthoretroviral LTR structure with three A-rich regions is reminiscent of structurally relaxed Giardia promoters. The five HMMs provided a novel broad range, repeat-independent, *ab initio* LTR detection, with prospects for greater generalisation, and insight into LTR structure, which may aid development of LTR-targeted pharmaceuticals.

## Introduction

Retroviral long terminal repeats (LTRs) are elaborate structures with important functions. They are the hallmark of the LTR retrotransposons which constitute from several percent to over half of many genomes. Understanding LTR structure and its evolution is a major problem in retrovirology and in genomics.

The retroviruses are grouped in seven genera: *alpha-, beta-, gamma-, delta-, epsilon-, lenti-* and *spumaretrovirus* [1]. Of these, the first six are classified as orthoretroviruses. Endogenous proviruses resembling the *beta*, *gamma* and *spuma* genera are common in vertebrates such as humans and mice. *Alpharetroviruses* have been found only in birds. *Epsilonretroviruses* have been predominantly detected in fish and amphibians. Endogenous *lentiviral* sequences were recently found in rabbits [2], whereas *deltaretroviruses* so far have no known endogenous counterparts.

LTRs contain regulatory sequences such as promoters, polyadenylation signals/sites and enhancers, and can therefore dramatically influence the RNA expression of both retroviral and nonretroviral sequences [3]. Vertebrate genomes contain thousands of endogenous retroviruses (ERVs) and, naturally, also thousands of LTRs. Single LTRs frequently form by excision of internal proviral structure after homologous recombination between the two proviral LTRs, identical at the time of provirus integration. Detection of single LTRs is a bioinformatical challenge and are difficult to align due to variable structures. Even more challenging is to find the common structure of LTRs and to understand their function. Part of the difficulty is that ERVs may be several 100 million years old [4] and have accumulated many deleterious mutations.

LTR recognition can be aided by the presence of target site duplications, and a few landmarks like AATAAA and TATA, together with promoter recognition algorithms [5], [6], [7], [8], but these features alone are too imprecise and prone to false positivity to allow genome wide searches. Paired LTRs in proviruses are less ambiguous. In fact, presence of two LTRs (formed identical during integration), separated by a characteristic distance, is one of the best ways to detect proviruses. The LTR_STRUC program [9], which is based on this method, is successful in identifying known families and discovering new families of LTR retrotransposons. Similar techniques are LTR_FINDER [10], a combined use of profile HMMs and other retrotransposon characteristics [11], [12], LTR_par [13] and LTRharvest [14]. However, in the human genome, most LTRs are present as single LTRs; they are about ten times more numerous [4] than proviral LTRs but cannot be detected by LTR_STRUC. A third means of LTR detection is based on their repetitiveness, i.e. occurrence several times in the genome. Their subsequent identification as retroviral LTRs rests on their occasional presence in proviral structures. The identification of the latter entails manual intervention and is not self evident. RepeatMasker (unpublished; see http://repeatmasker.org) searches for repeats, including single LTRs. RepeatMasker works against RepBase [15] which is a repeat database. The main drawbacks of RepeatMasker are that it is unable to detect low copy number LTRs, that it gives no information about their structure and that LTRs may be mislabelled as other kinds of repeats. Several other repeat-detecting new algorithms have been published, e.g. RECON [16], RepeatScout [17], PILER [18], and the use of multiple genomic alignments [19].

RetroTector^©^, a program package developed in our group [20] can detect endogenous retroviruses (ERVs) in genomic material in a repeat-independent way, and reconstruct LTRs and proteins. It uses the first two methods for detecting LTRs and is successful in identifying paired LTRs but less so in the case of single LTRs. During the work on RetroTector^©^, several features of LTR structure were identified, and incorporated into the LTRID program module. However, the sensitivity and specificity of these features were not enough for genome-wide analyses. We therefore had to find other pattern-recognition algorithms for single LTR detection. They are introduced in this paper.

LTR structure is highly variable. LTRs vary widely in length from a few hundred base pairs to over one thousand base pairs, and in structure. Among the vertebrate retroviruses, the longest LTRs occur in the *betaretroviruses*, e.g. MMTV and the HML elements, and the *spumaretroviruses*. LTRs comprise three regions, the U3 region which contains enhancer and promoter elements, the R region and the U5 region. Some of the long LTRs, like the MMTV LTRs, contain open reading frames (ORFs) which distort the basic LTR structure. The R region starts at the transcription start site (TSS) and ends at the polyadenylation site. LTRs usually contain a TATA-box located in the U3 region which however is missing or is distorted in some retroviruses, e.g. ERV9 [21]. Instead, ERV9 has two other promoter elements: One located upstream of the transcription start site, a GC/GT-box binding to the Sp1 transactivator protein, and an initiator motif (Inr) located at the transcription start site. The presence of a GC/GT-box (in inverted form) is also crucial for the promoter activity of HERV-H LTRs while their TATA-box may be dispensable [22], [23]. LTRs always have a polyadenylation signal, usually in the R region. This signal is most often the sequence AATAAA but ATTAAA and AGTAAA also occur. It is normally located 10–30 nt upstream of the polyadenylation site [24], [25]. However, in the case of the *betaretroviruslike* (http://www.ncbi.nlm.nih.gov/ICTVdb/Ictv/index.htm) human MMTV-like group 4 (HML4) [4], [26], the mRNAs were found to be polyadenylated 69 or 84 nt downstream of the AATAAA motif [27]. [27] found evidence for a stem-loop structure in R, which reduces the distance between the AATAAA motif and the polyadenylation site. Similar findings had previously been made for the *deltaretrovirus* HTLV-1 where the Rex responsive element (RexRE) forms a stem-loop structure [28]. In HTLV-1, the interaction between the RexRE and the Rex protein is essential for the regulation of expression of viral genes. *Deltaretroviruses* like HTLV have an AATAAA motif which comes before the TATA-box. The structural basis behind this anomaly is uncertain but folding back of U3 onto R may be the explanation (J Blomberg, unpublished) [28]. The R region is important for both transcription initiation and regulation of gene expression in other retroviruses as well. Examples are HIV [29], MLV and related retroviruses [30], [31], [32] and MMTV [33]. Surprisingly, transcripts promoted by HML2 LTRs were found to initiate close to the AATAAA motif which may therefore also function as a TATA-box [34] (Fig S1). Recent reports of antisense retroviral transcripts, promoted from the 3′LTR, in *gammaretrovirus-* and *betaretrovirus*like HERVs, as well as in HIV and HTLV [35], [36], [37], [38], [39], [40], [41], [42], [43], emphasise the need for a deeper understanding of LTR structure and function.

In the present study, we first improved the detection of single LTRs and second, learned more about their conserved structure. To do this, we used a mathematical tool, Hidden Markov Models (HMMs), Viterbi alignments [44] based on the HMMs, and proprietary heuristic algorithms to facilitate the modelling process and to remove false-positive sequences detected by the HMMs. The generality of LTR detection was assessed in several ways. One was the use of retroviral sequences from evolutionarily distant host genomes. For example, bony fishes, represented by zebrafish, diverged from other vertebrates around 400 million years ago [45], [46]. Birds, represented by chicken, diverged from other vertebrates around 300 million years ago [45], [46]. Marsupials, represented by opossum, diverged from placental mammals around 130 million years ago [47]. Although horisontal retroviral transfers of retroviruses between vertebrates have been common, see e.g. [1], these long periods of genetic separation in general correspond to a large difference of the ERVs of these species. The HMMs revealed LTR structural features common to several retroviral genera. Heterologous cross-genus HMM runs revealed the nucleotides responsible for the generalised LTR detection capability of some of the HMMs, which gave an insight into basic LTR structure, and, maybe, into basic LTR function. Although a completely general LTR detection was not achieved, several HMMs could detect LTRs in widely differing host species, showing that *ab initio* LTR detection was possible in a subset of LTRs.

## Results

HMMs are widely used in pattern recognition, e. g. in speech recognition [44]. The two key features of the model building is the *training* of the model whereby the HMM is taught which group of sequences it should recognise and the *evaluation* of the model whereby the HMM is tested on sequences belonging to the group of interest but not part of the training set. Five HMMs were built for five groupings of vertebrate orthoretroviruses: Betaretroviral HERVs or HMLs, general betaretroviral ERVs, gammaretroviral ERVs, lentiviruses and general vertebrate orthoretroviruses. The sequences of the training and evaluation sets were mainly RepBase consensus sequences [15], see Text S1, S2, S3, S4, S5.

We first explored the efficiency of the HMMs in detecting single LTRs in genomes, and then analyzed the HMM models to reveal conserved structures in LTRs.

### Testing HMMs for LTR detection in the human genome

As can be seen from Table 1, the average score of the training set varied from 45 for the most specialised lenti HMM to 5.5 for the broadest model, the general HMM. Each HMM was used to align the training set as a way to visualise the HMM. To test the detection capability of the model, human chromosome 19 (63 million base pairs) was screened. The chromosome was screened using a sliding window typically 1200 nt long with an overlap of 600 nt (the length of an average LTR). The log of the probability of each chunk was computed with the forward algorithm given the HMM. The scoring of sequences was done using the log odds ratio [48], which is the logarithm of the ratio between the probability given the HMM and the probability given a null model. The null model was identical to the HMM except that its match states had emission probabilities 0.25 for each base.

**Table 1:** Training set composition of the different HMM models.

Name of HMM	Test Set	Number of match states	Average length of LTRs in training set	Average score of training set	Number of LTRs in training set	Human beta	Mouse beta	Chicken alpha beta	Alpha	Lenti	Delta	Human gamma	Exogenous gamma
Hml	Jackknifing	170	728	40	23	23							
Gamma	Jackknifing	110	630	22	72							69	3
Beta	Lenti (8 sequences)	110	611	11	175	23	138	2	4		8		
Lenti	Jackknifing	190	386	45	37					37			
General	Lenti (8 sequences)	130	624	5.5	178	23	69	2	4		8	69	3

Preliminary runs indicated the presence of false positive hits coming from very CT-rich chunks. To reject them, a routine calculating the CT-content of each chunk was added. The highest CT-content in a sliding 100 nt window was determined. If it was higher than 80%, the chunk was considered as false positive because approximately 98% of the gamma- and betaretroviruslike LTRs had a CT-content less than 80%, corresponding to two standard deviations above the mean for a normal distribution. The statistics were performed on the training sets for betaretrovirus- and gammaretroviruslike LTRs. The result of the screening was compared with the RepeatMasker output for chromosome 19 of the human genome version Hg15 downloaded 2005/07/30.

A cross-correlation table between the LTR HMMs and RepeatMasker is shown in Table 2 and 3. The third, fourth and fifth columns are: The number of true positives (TP), i. e. positive by HMM and positive by RepeatMasker. The number of additional positives (AP), i. e. positive by HMM and negative by RepeatMasker. The number of false negatives (FN), negative by HMM but positive by RepeatMasker. The sensitivity is defined by the ratio TP/(TP+FN) and the specificity by the ratio TP/(TP+AP). This sensitivity and specificity are probably not the true ones. First of all, most detected retroviral groups were part of the HMM training sets meaning that detection may in part be due to overfitting. Furthermore, some retroviral groups in the studied genomes are heavily mutated, which makes it unrealistic to detect all members of such LTR groups. Last, RepeatMasker is not a perfect method. Thus, the calculated sensitivity and specificity only give an indication of the true values. To further evaluate the method, evaluation sets containing LTRs from opossum and other groups were used (see below).

**Table 2:** LTR HMM and RepeatMasker cross correlation at high specificity.

	Threshold	HMM+ REP+	HMM+ REP−	HMM− REP+	Sensitivity	Specificity	Number of hits on 63 M random sequence
Hml	5	395	18	57	0.87	0.96	<1
Gamma	5	391	159(20)	602	0.39	0.71	<1
Beta	7	146	12	313	0.32	0.92	<1
Lenti	3	2	14	1556	0.00	0.13	<1
General	4	102	88	1452	0.07	0.54	<1
Combined		804	276	719	0.53	0.74	-

The table shows the number of LTRs detected for different LTR HMMs as compared to the RepeatMasker output for LTRs of the same group, for chromosome 19 (63 million bp) of the human genome assembly hg15. The different thresholds were chosen so as to give roughly the same number of additional positives: 10–100. An algorithm for removal of CT-rich repeats was used, as described in *Results.* The number of false positives for runs of the five LTR HMMs on 63 million bp random sequence is shown in the last entry. The figure in parentheses is the number of non-ERV1 elements.

The betaretroviruslike HERVs consist of HML1 to HML10 [26]. The specialised HML HMM (“Hml” in Table 2 and 3) detected 87% of the HML LTRs detected by RepeatMasker in chromosome 19, with 96% specificity. The RepBase name was mapped to the corresponding HML group according to [4], [49]. The corresponding figures for the broader beta HMM (“Beta” in Table 2 and 3) were 32% and 92%, respectively. These results are dependent on the choice of the scoring threshold (see Table 2 and 3). Therefore, we show the sensitivity and specificity at two different thresholds for each model (Table 2 and 3). The thresholds were chosen so that the number of additional positives (found by the HMM but not by RepeatMasker) was roughly the same among the models (in Table 2 a few tens of them in chromosome 19 and in Table 3 around 1000), making a comparison between them easier. At the lower threshold 2 instead of 7 the sensitivity of the beta HMM increased to 68% at the price of a lower specificity: 28% (Table 3).

**Table 3:** LTR HMM and RepeatMasker cross correlation at low specificity.

	Threshold	HMM+ REP+	HMM+ REP−	HMM− REP+	Sensitivity	Specificity	Number of hits on 63 M random sequence
Hml	−4	403	1044	49	0.89	0.28	8
Gamma	−1	631	1051(653)	362	0.64	0.38	14
Beta	2	311	783	148	0.68	0.28	2
Lenti	−2	67	811	1491	0.04	0.08	18
General	1	423	1637	1131	0.27	0.21	<1
Combined		1080	4443	443	0.71	0.20	-

The table shows the number of LTRs detected for different LTR HMMs as compared to the RepeatMasker output for LTRs of the same group, for chromosome 19 (63 million bp) of the human genome assembly hg15. The different thresholds were chosen so as to give roughly the same number of additional positives: 1000. An algorithm for removal of CT-rich repeats was used, as described in *Results.* The number of false positives for runs of the five LTR HMMs on 63 million bp random sequence is shown in the last entry. The figure in parentheses is the number of non-ERV1 elements.

To check for the generality of the HML HMM, jackknifing was performed on the HMM training set and as can be seen in Table 4, the HMMs in the “HML HMM family” could detect most missing groups (excluded as part of the jackknifing scheme) in chromosome 19 with percent detection (of the missing groups alone) ranging from 6% to 88% and specificities around 90%, the exception being HML5.

**Table 4:** “Jackknifing” the HML HMM: Removing one group of the training set and detecting the group removed in chromosome 19.

Model	no_hml1	no_hml2	no_hml3	no_hml4	no_hml5	no_hml6	no_hml7	no_hml8	no_hml9	no_hml10
# match states	130	170	110	130	110	110	130	110	210	170
Threshold	5	5	6	5	5	5	6	5	6	5
Sensitivity (including removed group) (%)	60	87	62	78	70	82	80	74	87	87
Specificity (%)	91	96	93	95	91	91	91	93	86	91
% detection of removed hml group	52	88	6	83	0	59	71	23	67	67

The set of gammaretroviruslike HERVs can be divided into four main groups [1], [4]: *i.* The “HERV-E group”, also containing HERV-T and ERV3 [50]. This group is related to the exogenous retrovirus MLV and its relatives (the traditional gammaretroviruses) [1]. *ii.* The “HERV-I group”, also containing HERV-ADP. *iii.* The “ERV9 group”, also containing HERV-W, HUERSP3, MER41, MER66 and a few other groups [1], [51]. *iv.* The “HERV-H group”, also containing HERV-F [1], [52], [53]. At high stringency the gamma HMM detected 39% of the chromosome 19 LTRs belonging to the aforementioned groups and detected by RepeatMasker (see Table 2). The specificity was 71% corresponding to 159 additional positives. 139 of these additional positives were ERV1 elements according to the RepBase nomenclature [15]. The ERV1 elements are gammaretroviruslike [4]. Thus, these additional positives should not be considered as false positives. By decreasing the threshold from 5 to −1, the sensitivity and specificity changed to 64% and 38%, respectively (see Table 3). The generality of the gamma HMM was tested with the jackknifing technique and was found to be somewhat less than the HML HMM, see Table 5. There was no correlation between the age of ERV1 and ERV2 LTRs, as measured by the divergence between the 5′ LTR and the 3′ LTR (in chains containing both LTRs), and their detectability. Rather, the detectability depended on the LTR species, indicating that some orthoretroviral LTRs did not conform to the HMMs.

**Table 5:** “Jackknifing” the gamma HMM: Removing one group of the training set and detecting the group removed in chromosome 19.

Model	no_hervI	no_erv9	no_hervH	no_hervT
# match states	130	110	110	130
Threshold	5	5	5	5
Sensitivity (including removed group) %	38	35	34	43
Specificity (%)	75	70	72	73
% detection of removed gamma group	4.9	60	9.8	22

At a threshold of 4, the general LTR HMM, “general” in Table 2 and 3, had a sensitivity of 7% and a specificity of 54% compared to the RepeatMasker hits of HMLs, gamma- and spuma- like HERVs on human chromosome 19, while the corresponding figures at the lower threshold 1 were 27% and 21% respectively (Table 3).

The lenti HMM had as expected (because there are no known lentivirus ERVs in humans) low sensitivities at both thresholds (see Table 2 and 3) but did find 12 HML LTRs and 55 gammaretroviruslike LTRs at threshold −2.

To study the usefulness of HMMs as a general LTR screening tool, all models were combined (“Combined” in Table 2 and 3). At the higher thresholds, each of them equal to the threshold in Table 2, the sensitivity and specificity were 53% and 74% respectively while at the lower thresholds (Table 3) they were 71% and 20%, respectively.

In some models, “gamma”, “beta” and “general”, the nature of additional positives, found by the HMM but not by RepeatMasker, was investigated by using BLAT at the UCSC genome browser site. The ones that occurred as repeats, with at least 10 occurrences in the human genome, had a RepeatMasker hit such as a LINE or SINE elements [15], indicating that the more generalised HMMs occasionally detected these repeats.

The execution time on a computer with a 2.40 GHz dual processor and 2 GB RAM was about 2 hours per 10 million base pairs for an HMM with 100 match states.

### Comparison of HMM detection in random to actual genomic sequence

As shown in Table 2 and 3, all HMMs had a much lower positivity rate in random sequence than genomic sequence (represented by human chromosome 19). This illustrates the difficulty of obtaining adequate “non-LTR” control sequences.

### Testing HMMs for LTR detection in the opossum genome and other groups

The detection capability of all five models was tested on LTRs obtained from a RetroTector© run on the opossum genome (monDom4). This marsupial is separated from the human lineage by more than 100 million years, thus presenting a critical test on the detection range of the HMMs. The selected LTRs belonged to either the beta- or gammaretroviruslike chains according to the classification of RetroTector©. This machine-made grouping must however be regarded as provisional. Anyway, the current classification of retroviruses is largely based on those of the mouse. Vertebrate retroviruses are highly diverse. The mouse is relatively distant both from humans (another eutherian) and opossum (a marsupial). The nomenclature will need revision.

Table 6 and 7 show the sensitivities of all five HMMs on the set of beta and gamma opossum LTRs and ten other sets of LTRs: beta exogenous, HML consensus, alphabeta chicken [1], alpha exogenous, lenti, delta, spuma , epsilon, gamma exogenous and HERV gamma consensus. The results are shown in Table 6 and 7 for two different thresholds, the same as in the RepeatMasker comparison (Table 2 and 3).

**Table 6:** Sensitivities and average scores of the different HMMs for evolutionarily distant retroviral LTRs at high specificity.

	Thre-shold	Beta exo-genous	HML cons	Beta opossum	Alpha-beta chicken	Alpha exo-genous	Lenti	Delta	Spuma	Epsilon	Gamma exo-genous	HERV-gamma cons	Gamma opossum
Size of test set		3	23	89	47	3	8	8	7	4	3	69	474
Average length of LTRs		382	728	332	290	325	412	700	1177	809	554	630	448
Hml	5	−14	40*	−43	−49	−31	−11	−16	−15	−33	−20	−13	−34
		0	100%	1.1%	0	0	0	0	0	0	0	0	0
Gamma	5	−14	1.9	−13	−17	−23	−0.41	−0.14	−6.6	−8.2	12*	23*	−9.9
		0	22%	3.4%	0	0	2/8	1/8	0	0	3/3	97%	9.1%
Beta	7	2.3	18*	−5.8	1.5*	16*	3.7**	8.6*	1.8	−11	−8.1	2.1	−6.4
		1/3	100%	9.0%	32%	3/3	1/8	4/8	1/7	0	0	12%	1.9%
Lenti	3	−15	−5.2	−18	−17	−11	34*	−7.1	−14	−18	−7.8	−6.5	−16
		0	8.7%	0	0	0	100%	0	0	0	0	4.3%	3.0%
General	4	−2.2	9.4*	−5.9	−1.9*	5.0*	5.6**	3.0*	−1.1	−8.5	−3.6*	7.5*	−4.8
		1/3	96%	15%	30%	2/3	5/8	4/8	0	0	0	86%	10%

The thresholds were the same as in the RepeatMasker comparison in Table 2. (*) indicates that at least some LTRs in the LTR set are in the training set of the HMM model. (**) indicates that the set was used in the test set. The “beta”, “alphabeta”, “delta”, “spuma” and “gamma” categories contained ERVs and/or XRVs, further described in [1].

**Table 7:** Sensitivities and average scores of the different HMMs for evolutionarily distant retroviral LTRs at low specificity.

	Thre-shold	Beta exo-genous	HML cons	Beta opossum	Alpha-beta chicken	Alpha exo-genous	Lenti	Delta	Spuma	Epsilon	Gamma exo-genous	HERV-gamma cons	Gamma opossum
Size of test set		3	23	89	47	3	8	8	7	4	3	69	474
Average length of LTRs		382	728	332	290	325	412	700	1177	809	554	630	448
Hml	−4	−14	40*	−43	−49	−31	−11	−16	−15	−33	−20	−13	−34
		1/3	100%	7.9%	0	0	1/8	1/8	0	0	0	12%	1.7%
Gamma	−1	−14	1.9	−13	−17	−23	−0.41	−0.14	−6.6	−8.2	12*	23*	−9.9
		0	83%	20%	0	0	4/8	5/8	0	0	3/3	100%	22%
Beta	2	2.3	18*	−5.8	1.5*	16*	3.7**	8.6*	1.8	−11	−8.1	2.1	−6.4
		1/3	100%	18%	51%	3/3	6/8	7/8	3/7	0	0	49%	9.1%
Lenti	−2	−15	−5.2	−18	−17	−11	34*	−7.1	−14	−18	−7.8	−6.5	−16
		0	30%	6.7%	0	0	100%	1/8	0	0	0	23%	5.9%
General	1	−2.2	9.4*	−5.9	−1.9*	5.0*	5.6**	3.0*	−1.1	−8.5	−3.6*	7.5*	−4.8
		1/3	100%	28%	45%	3/3	7/8	5/8	1/7	0	0	96%	21%

The thresholds were the same as in the RepeatMasker comparison in Table 3. (*) indicates that at least some LTRs in the LTR set are in the training set of the HMM model. (**) indicates that the set was used as a test set.

Depending on the threshold, the gamma HMM detected 9.1% and 22% of the gamma opossum LTRs, and the beta HMM detected 9.0% and 18% of the beta opossum LTRs (Table 6 and 7). The general HMM had a similar detection capability for both groups. A dependency on LTR length was obvious. A higher frequency of opossum genome LTR detection was obtained with LTRs of similar length as those of the training set. Among 500–600 nt long opossum gamma LTRs, 66% were detected by the gamma HMM (Excel S1). A less dramatic effect of opossum LTR length was observed with the beta HMM. Around 60% of the opossum beta LTRs of 500–800 nt were detected by the beta HMM (Excel S1). The low scores of the opossum evaluation set depend on the presence of LTRs with aberrant LTR length.

HIV and other lentiviruses were most of them detected by both the beta and the general HMMs. Alpha RSV and most deltaretroviral HTLV:s were detected by the beta and general HMMs, but in this case they were part of the training set (see Table 7).

Thus, the HMMs had a degree of generality in detection of diverse LTRs which they were not trained for.

### Conserved structures of betaretroviruslike LTRs

**Figure 1:**
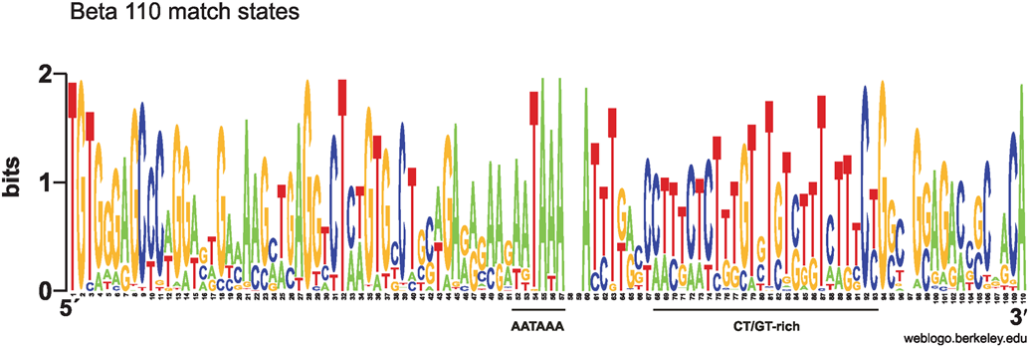
Weblogo for a Viterbi alignment of the beta training set (“beta” in Table 1). Insertions are not shown. The heights of the letters are a measure of how well conserved the residues are. Two bits correspond to 100% conservation.

The training set of the beta HMM with 110 match states, “beta” in Table 1 was Viterbi aligned and a sequence logo [54] was created from the alignment after the insert states had been removed. The most salient feature is the AATAAA motif at pos. 52–57 of the sequence logo (see Fig 1). No conserved TATA box is found. A GT-rich area is also apparent from position 75 to 91.

**Figure 2:**
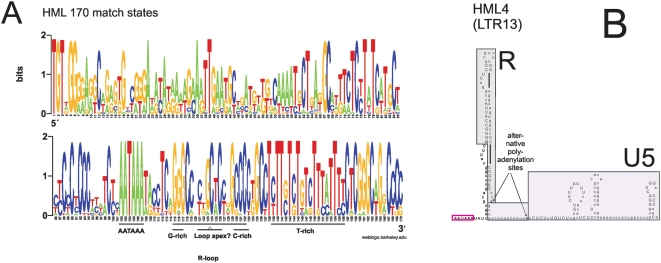
Outcome of the HML HMM. A. Weblogo for a Viterbi alignment of the HML training set (“hml”). Conventions are as in Fig 1. B. The HML4/LTR13 RU5 RNA sequence [55] analysed with the HML HMM, and folded with MFOLD (Fig S2a). Match state positions are in upper case. R boundaries, with alternative polyadenylation sites [55], are shown. AATAAA is boxed in red.

A sequence logo for the more restricted HML HMM with 170 match states is shown in Fig 2a. There is a well conserved AATAAA motif at pos. 102–107, but no TATA-box. Previous studies on HML2 have shown that transcripts may be promoted by the AATAAA motif [34] and that the putative TATA of HML4 is not conserved in an alignment containing five HML4:s from higher primates [55]. As in the beta case, a T-rich area is present, approximately at pos. 138–156. In addition, the weblogo has a conserved segment of guanosines (pos. 115–117) and after that, a conserved segment of cytidines (pos. 130–133). These were predicted to form a stem-loop structure (Fig 2b and Fig S2a), as presented earlier [27]. We investigated if the stem-loop structure predicted for HML4 was a general feature of the HML groups. For that purpose all 23 HML RepBase LTR consensuses were analysed with MFOLD in RNA mode. A similar stem-loop structure, probably situated in R, was predicted in 17 of them. It was stable in the sense that it was present in most alternative foldings. The conserved G:s base-paired with conserved C:s at the base of the predicted stem. The tip of the loop seemed to correspond approximately to pos. 121–126 in the HML HMM alignment. A few HML LTRs were not predicted to form these stem loops but this may be due to random post-integration mutations (Fig S2b). The predicted stem-loop structure could also be seen in the 170 nt consensus derived from the HML HMM (Fig S3). Two other stem-loops were often found in U5 but were mostly made up of an insert (relative to the HMM model), 40 nt long in average, located just before position 160.

As described by Colgan et al. [24], polyadenylation is dependent not only on the AATAAA motif but also on a T-, GT- or CT-rich area usually located 20–40 bases downstream of the polyadenylation site. This area is well conserved in the alignment (pos. 138–156).

### Conserved LTR structures of gammaretroviruslike HERVs

**Figure 3:**
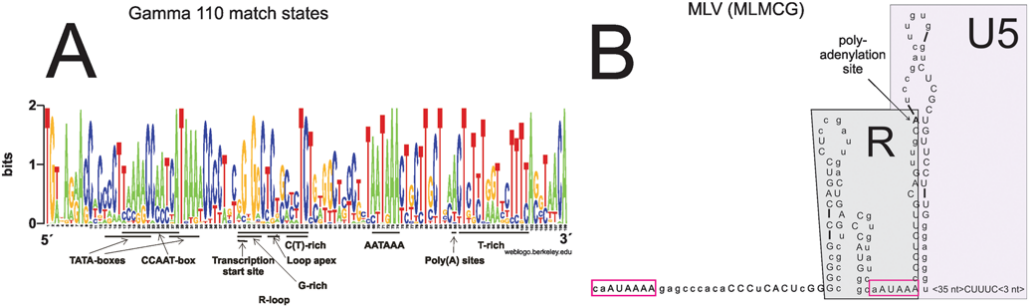
Outcome of the gamma HMM. A. Weblogo for a Viterbi alignment of the gamma training set (“gamma”). Conventions are as in Fig 1. B. MLV RU5 (MLMCG) RNA analysed with the gamma HMM and MFOLD. Match state positions are in upper case. R boundaries [43], [56], [57], [58], TATA box and AATAAA are shown. A partially conserved predicted stem loop early in R [31] is also shown.

A 109 nt consensus sequence was derived from the gamma LTR HMM. Transcription factor binding sites were sought with the MOTIF program. The consensus contains non-contiguous match states but this was taken into account by looking back at the Viterbi alignment of the training set. Only MOTIF matches which were contiguous were taken into account. The Viterbi alignment was visualised as a sequence logo (see Fig 3a). At position 87–101, a T-rich element is apparent. There is a clear AATAAA motif at position 69–74. Unlike HML LTRs, the distance between this box and the T-rich region is within the normal range (30–70 nt) [24]. Between them there are conserved A:s at position 85–86, probably poly(A) sites. For HERV-H, these putative poly(A) sites have been confirmed experimentally [56]. MOTIF predicts one TATA box at position 13–22 but another one is clear at position 27–32 (also found in the SuperViterbi alignment, see below). The second TATA box agrees well with experiments, for example in the case of gammaretroviruses (i.e. MLV and its relatives [57]), HERV-H [43] and HERV-I [58]. As mentioned in the introduction, ERV9 lacks a functional TATA box but has an AATAAA 28 nt upstream of the established transcription start site [21], which is *Inr* dependent. The transcription start site for HERV-H corresponds to GC/G at position 41–42 [43]. A conserved CCAAT-box between the TATA-boxes was also detected by MOTIF. The CCAAT-box is an upstream enhancer/promoter elements, common in vertebrate genes, recognised by the transcription factor NF-Y. It is located upstream of the TATA-box [59]. The found structure is consistent with the mammalian C-type LTR model of [6]. That model had a conserved hairpin loop in the R-region, also found in the MLV LTR (Fig 3b) by MFOLD (Fig S4). The gamma HMM consensus (Fig S5) also displays a shorter version of it. As in the HML case conserved G:s at position 41–45 bind to (less) conserved C(T):s at position 51–55 with the tip of the loop at position 47–49. This stem-loop has been studied in great detail for the MLV LTR [30], [31], [32] where it has been found important for RNA processing. As seen in fig 3b there are two other loops predicted in the R-U5 region of MLV.

### Conserved LTR structures of lentiviruses

**Figure 4:**
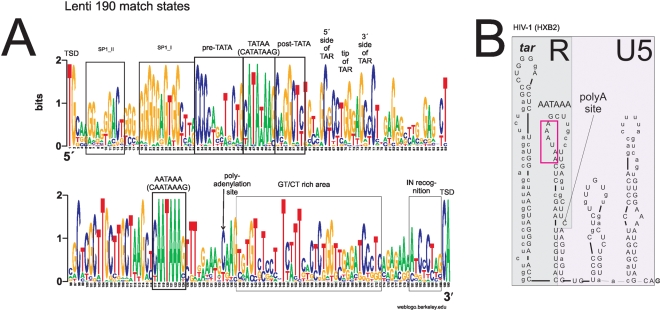
Outcome of the lenti HMM. A. Weblogo for a Viterbi alignment of the lentiviral LTR training set (“lenti”). Conventions are as in Fig 1. Conserved lentiviral landmarks (*nef* termination, TFBS and other characteristics of the proximal promoter), TATA and AATAAA boxes with their surroundings, TAR, the GT/CT rich stretch and a U5 portion which binds to integrase, are visible. B. RU5 of the HIV-1 hxb2 RNA sequence analysed with the lenti HMM. The conservation (upper case) of the crown and 3′ half of the *tar* stem loop, AATAAA and polyadenylation sites is shown.

The lenti HMM scored highly with all lentiviral LTRs. The structure of the lentivirus LTRs is qualitatively similar to that of the gammaretroviruslike LTRs. The most notable features of the 190 nt consensus (see weblogo Fig 4a) are a TATA-box detected by MOTIF at position 44–58 and an AATAAA-box at position 118–123. When the HIV hxb2 sequence was run with the lenti HMM, only a few landmarks before TATA proved to be conserved. From 5′to 3′, two of three Sp1 repeats (SP1_1 and SP1_2), as well as a “CCC” stretch, both part of the proximal promoter were conserved. Accordingly, when the consensus sequences were analysed with MOTIF, a conserved GC box element was detected at position 18–31. The GC-box is an upstream promoter element recognised by the transcription factor Sp1. MFOLD on the consensus predicted two stable stem-loops in the R-region, one corresponding to the *tar* loop at position 67–95, where in HIV1 the loop apex is at 68–73 (CTGGGA), and the remaining residues constituting the downstream part of the stem; they form base-pairs with inserts that can not be seen in the consensus. The second stem-loop comprises the region at position 97–142 with the AATAAA motif in the loop (Fig 4b; cf Fig S6). A GT-rich area is also found at position 136–164. Several of these lentiviral conserved features were are also found in a study [7] which used different bioinformatical methods, and a less diverse sequence set.

### Structures revealed by the general LTR HMM

**Figure 5:**
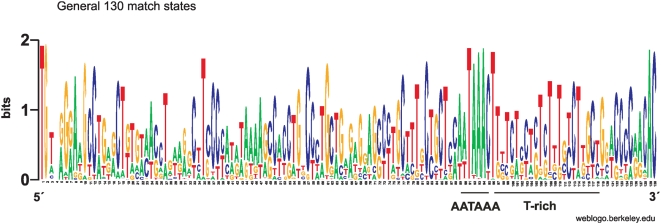
Weblogo for a Viterbi alignment of the training set containing both beta and gamma LTR sequences (“general”). Conventions are as in Fig 1.

The general LTR HMM with 130 match states, “general” in Table 1, generated the weblogo in Fig 5. As expected there is an AATAAA-box at pos. 88–105 and a less conserved TATA-box at position 39–48, both of them detected by MOTIF applied on the consensus. A conserved T-rich area can also be seen at position 106–114. Compared to the weblogos of beta, gamma and lenti LTRs the conservation is poor because it is the broadest model as can also be seen from the average score of the training set in Table 1. Nevertheless, some match states had dominating nucleotides which approached two bits in the weblogo.

### Combining Viterbi alignments of five different HMMs into a “SuperViterbi” alignment

**Figure 6:**
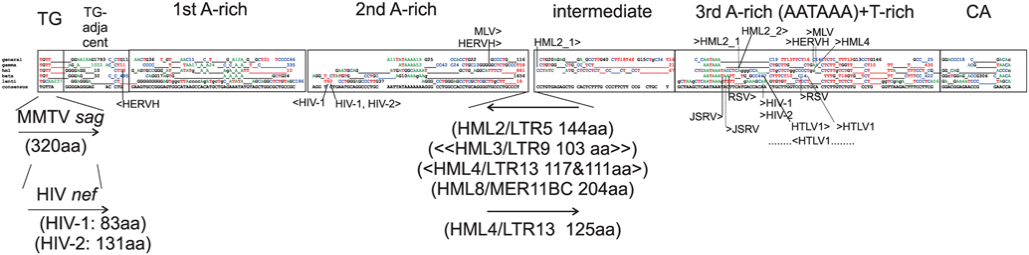
Combined alignment of Viterbi consensus sequences (“SuperViterbi” alignment) from the five HMMs. Each Viterbi is represented as a match state consensus sequence with the most common nucleotide, disregarding absence of fit. The SuperViterbi alignment was based on a Viterbi HMM alignment of the five Viterbi consensuses (Fig S7). In this secondary HMM, most primary match states were retained as match states (upper case). A few became insert states (lower case). Insert state figures from each Viterbi consensus were then added. Insert state numbers above 20 were given an additional space to denote a continuity break. Insert states occurred nonrandomly. Taking them, together with conspicuous and previously known motifs like TG, TATA, AATAAA, T-rich and CA, into account, modules could be discerned after conservative manual sliding in the original SuperViterbi alignment. The origins of the HMMs are further clarified in Table 1. A consensus based on the manually adjusted SuperViterbi alignment is shown below the other sequences. Landmarks derived from well characterized LTRs were plotted in the manually adjusted SuperViterbi alignment. Transcriptional start sites are shown as full lines, where viral abbreviations were followed by “>” if in sense and by “<” if in antisense direction. Polyadenylation sites were shown by dotted lines, with viral abbreviation preceded by “>”. Abbreviations and accession numbers: TSS and polyadenylation sites are shown for HIV-1 (strain hxb2), HIV-2 (D00835), HML2_1 [79] , or alternatively HML2_2 [34] , HML4 [27], [55], JSRV (AF357971), HERV-H [23], MLV (J01998), RSV (NC_001407) and HTLV-1 (HL1PROP). TSSs and polyadenylation sites were mapped onto the beta HMM consensus except HERV-H and MLV which were mapped on the gamma HMM consensus. Positions of ORFs are also given. HIV-1 *nef* (MNCG) reaches into around half of U3. The HML ORFs, found in RepBase or Clustal LTR consensus sequences, are either contained between the second A-rich and intermediate modules, or overlap with several modules (HML3). They are antisense to the standard retroviral transcriptional sense, except for one (HML4). The module-overlapping HML3 ORF is symbolized with “<<..>>”.

**Figure 7:**
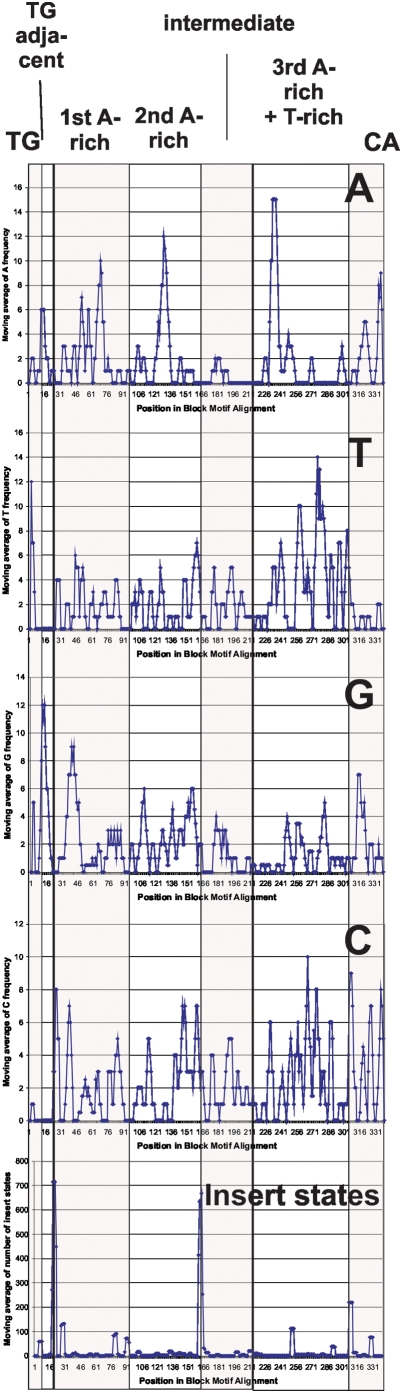
Distribution of nucleotide frequency and insert state length per position in the SuperViterbi consensus alignment (also referred to as “block motif alignment”). A moving average over a window of 4 alignment positions was used. The structural basis for the seven modules is shown by this plot. Insert states stretches are longest at the ends of the Viterbi consensus sequences.

During the work with the Viterbi alignments for each HMM, evidence for a common orthoretroviral LTR structure emerged. Insert states were not randomly distributed. They tended to occur in contiguous stretches at certain relative positions in the Viterbi alignments. This, and the previously mentioned conservation of characteristic contiguous nucleotide stretches, sometimes appreciable as “motifs” in the match states, made it possible to align them in a combined Viterbi alignment. Initially, an HMM was trained on the five HMM consensuses. A preliminary “SuperViterbi” alignment resulted (Fig S7). This alignment was then manually adjusted, taking into account the distribution of insert states, nucleotide composition and the presence of known motifs (Fig 6 and Fig 7). Seven modules with small internal and longer intermodule insert states could be discerned, 1. “TG”, 2. “TG-adjacent”, 3. “first A-rich”, 4. “second A-rich”, 5. intermediate, 6. “third A-rich+T-rich” and 7. “CA”. The discrete distribution of the four nucleotides and insert states within the alignment of five HMM consensus sequences is visible in Fig 7. It supports that orthoretroviral LTRs consist of modules. The modules, or variants of them, have appeared in all of several hundred LTR HMMs of differing number of match states throughout this work (data not shown).

The most conspicuous feature was the almost universal occurrence of a TG at the 5′ end, and a frequent occurrence of CA at the 3′end. These inverted repeats are characteristic of vertebrate retroviral LTRs [29]. Their inclusion in the Viterbi alignments serves as a quality control. The other modules are discussed under the respective LTR region below.

As seen in Fig 6 and mentioned below, the R boundaries, transcriptional start and polyadenylation site, do not precisely match the conserved modules. However, U3 is the least conserved of the three regions, and contains proportionally fewer match states than R and U5.

1. The initial, almost universal, “TG” module was followed by 0-237 insert states. The long insert state in some lentiviruses is mainly due to the *nef* ORF. 2. The “TG-adjacent” module contained two short portions. The first contained combinations of “GG” and “GA”, the second of “CC” and “CT”, and 0 to 1793 nt insert states. 3. The “first A-rich” module consisted of stretches of up to four A:s, followed by a region of “C”, “TG” and “C”. This may correspond to the noncanonical TATA which sometimes occurs upstream of the canonical TATAA [7] in gammaretroviruses. In lentiviruses, the TATA box, is present in this module. It was placed there by the HMM alignment of HMM consensus sequences (Fig S7). On inspection, this is reasonable because of sequence similarity to the modules of the other four HMM consensuses. It ends with 10-335 insert state stretches. 4. The “second A-rich” module contained a more or less clear TATA box in all except the “beta” and “lenti” HMM consensuses. The A-rich stretch is followed by G and C repeats, and ends with 1-4 T and 18-1656 insert states. Especially long insert state stretches occurred in the HML HMMs, and contained long (>97 aa) ORFs in the HML2, HML3, HML4 and HML8 LTR consensus sequences, illustrating that long ORFs are especially common in betaretroviruslike ERV LTRs (which include *sag* of MMTV; data not shown). Except for one of the HML3 ORFs, which encompasses also a part of the intermediate module, all observed HML ORFs start or end in the intersection of the second A-rich and intermediate modules. These ORFs are further discussed below. 5. The “intermediate” module was “CT” rich and ended with 16-67 insert states. It was not present in the beta and lenti HMM consensuses. 6. The third A-rich module, situated in R-U5, encompassed the AATAAA box. It started with “AATAAA” in different embodiments, directly followed by a CT/GT rich region which contains the polyadenylation site, and ends with 0-430 insert states. Match states could be mapped onto stem loops in this region (see above). 7. Finally, the “CA” module started with combinations of “GG” and “CC” and in three of five cases ended with “CA”, the canonical target site duplication. In fact, “CA” was present in nearly all of the LTRs on which the HMMs were based. However, the HMMs did not always pick it up as a majority consensus, as can be seen in the web logos.

### Fit of exogenous retroviral LTRs in the respective HMMs

The structure and function of alpharetroviral (ALV), betaretroviral (MMTV, MPMV and JSRV), gammaretroviral (MLV) and lentiviral (HIV) LTRs are especially well known [60]. The degree of fit of well characterised exogenous retroviral LTRs, occurring in the last three of the four genera, to the HMMs (which were largely based on ERV LTRs) was therefore of interest. The exogenous MMTV (AF033807) and MPMV (AF033815) LTRs fitted to some extent (scores of -4.0 and -7.9, respectively) in the beta HMM, while JSRV (AF357971) fitted well (score 10.3). The exogenous MLV-like sequences (MLV J01998, GaLV M26927 and FLV M18247) fitted (with scores 8.2, 13 and 14, respectively) in the gamma HMM in the second A-rich module: These HMMs thus encompassed many endo- and exogenous beta- and gammaretroviruslike sequences, even if their generality for opossum counterparts with aberrant LTR length seemed relatively weak.

The lenti HMM detected all lentiviral LTRs, including Visna (score 25), EIAV (score 15) and the rather aberrant RELIK (score 12) [2] LTRs. RELIK was not part of the training set for lenti LTRs. This attests to the fidelity of the lenti HMM for lentiviral sequences. Its HMM consensus was mainly based on *nef*-pruned primate lentiviral LTRs, and did not yield “second A-rich” and “intermediate” modules (nr 4 and 5). The pruning, necessary to achieve convergence during HMM training, may have contributed to this lack of two modules. However, the first and third A-rich modules including a short CT rich stretch, were obvious.

### Evidence for a redundant basic LTR design

The three A-rich modules have common features. The A-dense islands are 5–15 nt long, surrounded by “CC”, “CT” and “GT” rich stretches. The consensus TATA and AATAAA sequences are often strikingly similar. In the lentiviral HMM consensus they are conserved within “CATATAAAG” and “CAATAAAG” contiguous match state stretches, respectively, differing only by an inserted T. Lentiviral LTRs have only two A-rich modules. Their TATA consensus maps to the first A-rich module, and they lack the second A-rich module. It has been noted by others [8] that gammaretrovirus LTRs have a TATA-like sequence upstream of the “regular” TATA box. Another sign of redundancy is that AATAAA functions as TATA in HML2 [34]. A conserved structural feature is a stem loop with a UG-rich crown, corresponding to *tar* in HIV-1, just after the transcription start site (TSS), see Fig 2, 3 and 4. The polyadenylation signal (AATAAA) may or may not be on a second stem loop. The polyadenylation site is situated 10–30 nucleotides after AATAAA, in the first half of the CT/GT rich stretch which invariably follows after AATAAA.

### Mapping of sense and antisense transcripts onto the HMM LTR consensuses

At least three retroviruses are now known to produce antisense transcripts [35], [36], [37], [38], [39], [40], [41], [42], [43]. Some of these initiate within the 3′ LTR. We therefore mapped the antisense TSS onto the SuperViterbi alignment. These sites were variable in position (Fig 6). In HIV and HTLV sense and antisense TSSs were relatively close to each other. In HERV-H, they were far from each other.

### Cross-genus recognition by the HMMs

When the HMMs were tested against LTRs from retroviral genera which they were not trained for, the general pattern was that the TG, second and third A-rich, the T-rich and CA modules most frequently contained match states with conserved nucleotides (data not shown).

The degree of crossreactivity of the HMMs tended to follow the degree of relatedness of the Pol sequences of the corresponding proviruses [1]. The gamma HMM was the most cross-reactive (Table 7). It detected 83% of the HML consensus LTRs, 4/8 of the lenti- and 5/8 of the deltaretroviral sequences. The beta HMM detected 49% of the gamma HERVs (“ERV1” RepBase consensus sequences), and 3/7 of the spumaretroviruses (“ERVL” or “ERV3” in the RepBase notation). The aberrant primary structure of deltaretroviral LTRs [60] could be a reason why deltaretroviral LTRs gave weak cross-genus scores and weak cross-genus Viterbi alignments. However, the general structural pattern of cross-genus recognition is a further sign of the generality of the HMMs.

### Position of known and putative ORFs in the combined Viterbi alignment

**Figure 8:**
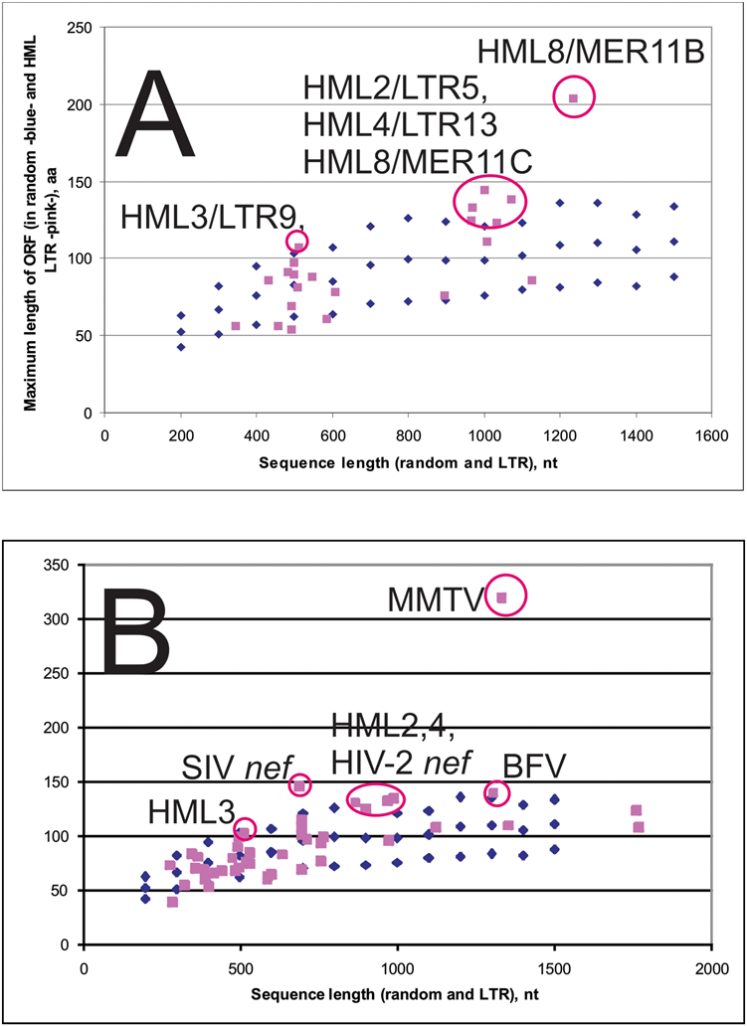
Plot of the longest ORF detected in LTRs selected from all seven retroviral genera. ORFs longer than 97 amino acids were detected in MMTV *sag*, primate lentiviral *nef*, Bovine Foamy virus (BFV), and HML2,3,4 and 8 consensus LTRs. One standard deviation of the longest ORF occurring in 100 random sequences of increasing length is also shown. A. Longest ORF in RepBase consensus HML LTRs. B. Longest ORF in Clustal consensuses of alignable HML LTR groups and other LTRs (45 in total). Sequence identities and other details are given in Excel S2.

The occurrence of open reading frames longer than 100 amino acids in LTRs of 500–1000 nt is at the fringe of likelihood. A likelihood fringe of one standard deviation was calculated in a simulated set of random sequences of different length (100 random sequences for each length in increments of 100, from 200 nt to 1500 nt). Open reading frames outside of the likelihood fringe occurred in some of the LTRs (Fig 8; Excel S2) The 5′ third of primate lentiviral LTRs had a long ORF, encoding the *nef* protein. MMTV had the long *sag* ORF in the same position. Several of the long HML group consensus LTRs (HML2/LTR5, HML3/LTR9, HML4/LTR13 and HML8/MER11B/MER11C) harboured antisense ORFs (see Excel S2) which had a length exceeding one standard deviation of the longest ORF length per sequence in the random sequence set. MMTV *sag*, HIV/SIV *nef* and HML8 ORF were clearly outside the random zone. The HML ORFs were close to the 1 SD border. However, compared to other LTRs, HMLs were more often outside of the 1 SD border (Fig 8). None of the HML ORFs started with a methionine, which would have been expected. However, most of them were situated at the interface between the second A-rich block and the intermediate module. However, an HML3 ORF also overlapped with the intermediate module. If these ORFs were nonfunctional, occurring by chance, they should have occurred in random positions of the Viterbi alignments. The HML4 consensus sequence was remarkable in that it contained three ORFs longer than 100 amino acids, all situated at the abovementioned interface.

### Projecting transcription factor binding sites (TFBSs) onto the Combined Viterbi alignment

TFBSs are best known for ASLV, MLV, HIV and MMTV. They were often hard to identify in the SuperViterbi alignment. However, a CCAAT just before the second A-rich domain was conserved in the gamma HMM. TFBSs are adaptive stuctures which change due to requirements for tissue specificity and pathogenicity. When LTRs of entire genera were Viterbi aligned, they were often not conserved and occurred as insert states. However, the lenti HMM had conserved specificity protein 1 (SP1) TFBSs.

## Discussion

Detection *ab initio* of single LTRs in genome databases is an important bioinformatical goal. In this work, a wide variety of vertebrate retroviral LTRs was investigated. However, orthoretroviruses, comprising the alpha-, beta-, gamma-, delta-, epsilon- and lentiretroviral genera, were the main target of our efforts. Nevertheless, epsilonretroviruses generally got low scores. Neither were attempts to include spuma-, erranti- and pseudoretroviral LTRs in training and evaluation sets successful. However, the training and evaluation LTR sets were gathered from evolutionarily very diverse hosts. Without the knowledge that they all were LTRs from vertebrate proviruses it would have been impossible to demonstrate a common structure in them. In this sense, the common structures demonstrated by the Viterbi alignments were a significant achievement. Using them as a base, it might be possible to extend the generality of LTR detection further.

We used HMMs to detect and align LTRs from mainly beta-, gamma- and lentiretroviruslike RVs. The results show that the problem of detecting and aligning single LTRs can be solved for at least certain kinds of LTRs. The outcome of runs against a variety of vertebrate ERV LTRs, and the results of jackknifing, indicate a considerable generality in the detected LTR structure.

There were two drawbacks with the LTR detection. First, our HMMs sometimes did not find groups not present in the training set. For example, a beta HMM not trained for HML5 LTRs would not detect them. It seems that LTRs are too variable in structure to allow a complete generalisation with the chosen technique. It is also expected that postintegrational mutations will distort some of the structures. Second, the computation speed was quite low on a standard computer.

Common to all HMMs was that random sequence had a much lower positivity rate than actual genomic sequence (which was used to calculate specificity). Thus, there seems to exist a basic “LTR-like” character of vertebrate genomic sequence that is not present in purely random sequence. The reason could be that genomes were largely built from transposons more or less related to LTRs, or that LTR trained HMMs have a propensity to react with common genomic stuctures like promoters and enhancers. A similar difference between random and genomic sequence was found during evaluation of promoter recognition algorithms see e.g. [61].

The most conserved structures, common for all five HMMs, are TG, the AATAAA motif, the T-rich element and CA. The same tendency was seen in the cross-genus runs. The TATA-box is well conserved in gamma and lenti LTRs but less so in beta LTRs.

The Viterbi alignments were compared with Clustal alignments (not shown). The main difference between them was that the conserved motifs were displaced in Clustal alignments and as a result it was almost impossible to construct a reliable consensus sequence. Clustal alignments are also more sensitive to the number and choice of sequences. For example, if many post-integrationally disrupted sequences are present, or sequences with a variable amount of inserts, they can destroy the alignment. To a degree, HMMs seem better at extracting information from such sequences.

We found evidence for a stem-loop in the RU5 region of the human beta (HML) LTRs, reducing the unusually large distance (up to 60 nucleotides) between the poly(A) signal and the T-rich segment, which contains the polyadenylation site. Since the AATAAA motif can function as a TATA-box for HML2 [34] we speculate that this stem-loop plays a role in transcriptional activity as well. Although TATA normally acts at the DNA level and AATAAA at the RNA level, they seem to have a functional flexibility where the former may substitute for the latter. Stem-loops in the beginning of R were predicted also in gamma and lentiviruses, in accordance with previous models [29], [31]. The Viterbi consensus of the five HMMs did not generally include the loop stems. Although the stem-loop secondary structure may be conserved, the corresponding primary structure, on which the HMMs work, is not as conserved.

The existence of retroviral antisense transcripts has been proven for at least three viruses: HIV-1, HTLV-1 and HERV-H [35], [36], [43]. So far, there is no consensus regarding their start sites. We therefore plotted some of the known start sites in the 3′ LTR in the SuperViterbi LTR model. Both HIV and HTLV antisense transcripts were reported to start close or relatively close to the respective sense TSS. However, antisense HERV-H starts at a different LTR location than the HERV-H sense transcript.

Sense and antisense ORFs were found in consensus sequences of LTRs from certain betaretroviruslike ERVs; HML2, HML3, HML4 and HML8. HML LTRs are 500–1000 nt long, longer than most other LTRs, but shorter than the MMTV LTR, which is 1300 nt, and contains the *sag* ORF. *Nef* ORFs occur in some primate lentiviral LTRs. Otherwise we did not find ORFs of the same length in LTRs of other orthoretroviruses. The HML group consensus sequences are probably close to the proviral sequence as it was just after integration. The translated amino acid sequences of the ORFs (see Excel S2) did not start with a methionine, which would have been expected. The sequences did not have closely matching known proteins in GenBank. Neither were they similar to each other. This could be a sign of independent acquisition by the respective retroviruses. When the long HML8 ORFs were used in a search against RefSeq cDNA sequences, several interesting transcripts were found: *i.* The Xist sense-antisense pair which regulates epigenetic X chromosome silencing [62], [63] and *ii.* a transcript overlapping the APOBEC3B gene in antisense were found (Fig S8). However, the HML8 sequence probably did not encode a functional protein in these transcripts because there was no long ORF where the HML8 ORF fitted in. This fits with the reported loss of ORF and accumulation of transposon repeats during the evolution of XIST [62]. Moreover the HML8 ORF was broken by a stop mutation in both transcripts. Incidentally, the transcript which overlaps the APOBEC3B gene on chromosome 22 starts in the HML8/MER11C LTR very close to the start site found in HML2 (Fig S1). Although out of scope for his paper, we cannot resist mentioning that it should be considered whether HML8 can somehow participate in epigenetic silencing at least at these two loci. Maybe a retroviral promoter (HML8) controls the expression of the antiretroviral gene APOBEC3B. Apart from this, the putative HML ORFs deserve a further study.

In the well studied HIV and MLV cases, it was possible to project known enhancer and promoter sequences onto the combined Viterbi alignment. Conserved lentiviral landmarks were the SP1 transcription factor binding sequences. The gammaretrovirus HMM homogeneously detected most gammaretroviruslike sequences, and probably gives an accurate representation of conserved gammaretrovirus LTR structure. Preceding the second A-rich stretch was a partially conserved CCAAT (CAAT box). Otherwise, transcription factor binding sites in U3 were not well conserved in the HMM consensus sequences.

With the experience from orthoretrovirus-directed HMMs, it should be possible to tackle LTRs of other retroviruses, and LTR transposons such as the *Meta-* and *Errantiviridae*, i.e. Ty1/copia and Ty3/gypsy elements, respectively. They did not score highly with the present HMMs, but it is likely that more or less specialised HMMs could be built from them also.

Although at least two A-rich domains occur in the Viterbi alignments of all five HMMs, their exact sequence is variable. To some extent they can substitute for each other. Indeed, TATA and AATAAA box consensuses tend to be similar. This variable and redundant structure and function is reminiscent of the relaxed stuctural requirements for promoter structure in some protists, like Giardia [64], where A-rich domains can serve as bidirectional promoters in a flexible fashion. LTRs seem to retain some of this flexibility, and thus may reveal an original basic RNA polymerase II promoter organisation.

Current knowledge of LTR structure and function is becoming more detailed, but also less definite than previously appreciated [60]. For example, bidirectionality of transcription, and variability of transcriptional start sites, even in ordinary sense transcripts, have recently been reported [35], [36], [37], [38], [39], [40], [41], [42], [43]. The suggestive evidence for a modular LTR structure presented here, and previously by others [6], [7], may provide a basis for a more fundamental understanding of LTR function, and for construction of more general LTR-recognition algorithms.

A better understanding of LTR structure will shed light on changes in retroviral tissue specificity and pathogenicity [60], on how bidirectional promoters and open reading frames [65] can be accommodated in their structure and on how LTRs can serve as alternative promoters in vertebrate genomes [3]. In addition, LTRs can spawn highly variable individual-specific minisatellite sequences [66], [67], [68], [69]. Knowledge of LTR structure could improve the understanding of this process.

In summary, the HMMs were able to detect single LTRs in the human genome, without reference to cognate complete proviruses or repetitiveness. The sensitivity and specificity were high for some of them. They also provided alignments encompassing LTRs of most of beta-, gamma- and lentiretroviruslike LTRs, presenting further insight into the common LTR structure of these genera. This can have implications for gene therapy, LTR-based antivirals and for the understanding of both retroviral and vertebrate genomic evolution.

## Materials and Methods

### LTR sequences for training, testing and evaluation

The training sets consisted mainly of endogenous LTR consensuses retrieved from RepBase [15]: 23 HML LTRs, 69 gammaretroviruslike human ERV1 LTRs selected according to [4] from a set of 198 human ERV1 sequences and 138 betaretroviruslike ERV2 mouse consensus sequences. Primate lentiviral LTRs were obtained from “HIV Databases”: http://www.hiv.lanl.gov/. LTRs from genomewide RetroTector© analyses of the opossum genome version monDom4 and the chicken genome version galGal3 were used mainly in the evaluation sets. LTRs from proviruses with two LTRs, scoring higher than 500 in RetroTector©, were used. This insured that the LTRs were authentic, with retained structure. Some reference exogenous and endogenous LTRs from all seven genera, downloaded from GenBank were also collected. Genome sequences were downloaded from the UCSC ftp site. The training, test and evaluation sets are described in Table 1, 4, 5, 6 and 7 for each HMM. The detection capability of the HMMs in large genome sequences was tested against human chromosome 19 of the Hg15 assembly.

### HMM algorithms

Algorithms such as Clustal have difficulties in aligning sequences that differ much from each other and lack easily detectable structure. Since LTRs are of this kind [3], we decided to work with HMMs. HMMs are probabilistic models which precisely can represent the degree and sequence of interdependence of a series of states. As described below, we trained several hundred HMMs, separately for each retroviral genus, or more general ones, using different training sets and training parameters. Each HMM was evaluated on sets of independent LTRs. The HMMs were used to screen genome sequences with the ultimate goal to find previously undetected single LTRs. This is demanding, however, as in many cases there will be no way to verify such newly detected single LTRs. Beside the detection aspect, the HMM models can be analyzed to gain insight into LTR structure. We used a so-called profile HMM, an architecture introduced by Krogh et al [70]. Profile HMMs can construct and represent nucleotide alignments. As any HMM, they consist of interconnected states. A profile HMM has a number of modules, corresponding to the conserved columns in the alignment. Each module contains a *match* state, an *insertion* state to allow for insertions and a *delete* state to handle gaps.

The three basic tasks for HMMs [44] are: 1. Given a family of sequences, to construct the HMM which best represents the family. This is a statistical estimation problem for which the standard method used is a maximum likelihood estimate computed by an algorithm known as the Baum-Welch algorithm [48]. 2. To calculate the probability that a given HMM will generate a given sequence. This is done by a dynamic programming algorithm called the forward algorithm. 3. To align one or several sequences against a given HMM or equivalently, determine the highest probability path(s) through the HMM. This is solved by the Viterbi algorithm and the resulting alignment is called a Viterbi alignment.

### Building HMMs specific for LTRs

There are two steps in building an HMM, initialisation and training. The parameters of the HMM can be initialised by using a single sequence or a pre-existing alignment. The main step is training: A set of sequences is used to optimise the parameters of the HMM. One difficulty with training an HMM is to design a good training set. If there are many sequences that are very closely related to each other, the HMM will become overspecialised to this group. One way of circumventing this pitfall is to replace the group of closely related sequences with their consensus. Another way is sequence weighting of which there are several variants. In this work the maximum entropy weighting method [71] and manual weighting were used together with the first method. The most serious difficulty with an HMM model is overfitting, which means that it represents the training sequences well but fails to generalise to related sequences not present in the training set. To reduce overfitting, a maximum *a posteriori* (MAP) estimation algorithm was used besides the maximum likelihood based Baum-Welch algorithm. The MAP estimation algorithm in this work is from Brand [72]. It uses a so called entropic prior which has a parameter *z* that controls the order (if positive) or disorder (if negative) of the HMM model. In general the somewhat disordered or noisy HMMs had higher generalising capacity.

Many HMMs (an HMM “family”) with different number of match states and different *z* were constructed for the gammaretroviruslike genus of LTRs. An epsilonretroviral LTR sequence closely related to the gamma genus, WDSV (accession number AF033822), was chosen to initialise the HMMs. The training set contained 72 sequences: 69 Repbase human ERV1 LTR consensuses and three exogenous gamma LTRs.

Similarly, two HMM families designed to cover mainly the beta genus were constructed. One HMM family was specialised to the HML group of betaretroviruslike LTRs. Its training set contained 23 human ERV2 or HML LTR consensuses. The other HMM family was broader in scope: Its training set consisted of LTRs from five groups (see Table 1): 1) The same 23 HML LTR consensuses. 2) 138 mouse ERV2 LTR consensuses. 3) Two intermediate beta LTR consensuses constructed from a RetroTector© analysis of the chicken genome. 4) Three alpha exogenous LTRs and one endogenous alpha LTR also from the chicken genome. 5) 8 delta exogenous LTRs. The initialising sequence were in both cases a BLV LTR which belongs to the delta genus (accession number K02120). Because of the varying number of sequences in each group manual weighting was performed.

A purely lentiviral HMM family, with training set consisting of 32 primate lentiviral LTRs plus five lentiviral LTRs from other mammals, was built in the same way. Three of the 32 primate LTRs were CLUSTAL [73] consensuses. Manual weighting was done due to the overrepresentation of primate LTRs and it was found necessary to remove the long ORFs present in the primate LTRs (*nef*) to achieve convergence.

One general HMM family was also built. The initialising sequence was a BLV LTR as before and its training set was a combination of the gamma and the broader beta training sets (see Table 1).

The initialisation gave an HMM with number of match states equal to the length of the initialisation sequence, around 500 for a typical LTR. Since the probable number of conserved sites is much lower than this, model surgery [48] was applied during the training to reduce the number of match states. Another reason for reducing the number of match states is to save computation time which increases linearly with the number of match states. Finally, by reducing the number of match states, the number of free parameters in the HMM is decreased, which counteracts overfitting. The choice of initiation sequence was not so critical as long as it was not too short.

To choose the best model among the HMMs with varying number of match states and different *z*-values, test sets were used and the model giving the highest score for this test set was selected. In some cases (see Table 1) the test set was just a family of sequences not contained in the training set and in other cases “jackknifing” was performed (see Table 4 and 5), i. e. removing one family at the time from the training set and calculating the score of the family removed. It was found that the score of the test set increased linearly with the number of match states in the HMM model until it reached a plateau. The “best” HMM model was chosen at the end of the linear regime or beginning of the plateau. Generally the selected models had 100–200 match states and negative *z*-values.

### Software

Several bioinformatical tools were used in this work: RetroTector© [20], as described above. Mega version 3.1 [74] and ClustalX [75] were used for alignments and phylogenetic analyses. Bioedit [76] for viewing alignments. Mfold [77] for prediction of nucleic acid secondary structures. MOTIF [78] for finding transcription factor binding sites. The RepeatMasker output downloaded from the UCSC genome ftp site (unpublished; see http://repeatmasker.org). Weblogo was used for constructing sequence logos [54].

To implement HMMs, we used programs written in C. These were mainly modules implementing the three basic tasks for HMMs as described above. To these, a few modules were added: A module for Viterbi alignments, a module for weighting sequences using the maximum entropy method, a module for adjusting the length of HMMs and a module for regularising HMMs based on [72]. These programs were applied to the problem of LTR detection and characterisation.

## Supporting Information

Figure S1 Start sites of some HML transcripts, including one from the ABOBEC3B locus on chromosome 22.
(2.38 MB TIF)

Figure S2 Conformational analysis of the HML4 and HML5 RU5 regions. The MFOLD package was used.
(2.90 MB TIF)

Figure S3 Conformational analysis of the HMM generated HML consensus. The MFOLD program was used.
(2.17 MB TIF)

Figure S4 Conformational analysis of RU5 of Murine Leukemia virus (Accession nr MLMCG). The MFOLD program was used.
(2.51 MB TIF)

Figure S5 Conformational analysis of the gamma HMM generated LTR consensus sequence. MFOLD was used.
(2.10 MB TIF)

Figure S6 Conformational analysis of RU5 of HIV-1 LAV LTR. MFOLD was used.
(2.84 MB TIF)

Figure S7 The Viterbi alignment of five HMM consensus sequences. This HMM-generated “superviterbi” alignment was manually adjusted with regard to insert state clustering and presented in figure 6.
(3.25 MB TIF)

Figure S8 BLAT result of a search with HML8 LTR ORF in the hg18 assembly. An antisense HML8 LTR was found in the APOBEC3B locus on chromosome 22.
(9.34 MB TIF)

Excel S1 The figures illustrate a great dependence on LTR length when the beta and gamma HMMs are applied to opossum LTRs.
(0.66 MB XLS)

Excel S2 Sequences of the ORFs of human MMTV-like LTRs, and their positions in them.
(0.16 MB XLS)

Text S1 HML training set
(0.01 MB TXT)

Text S2 Beta training set
(0.31 MB TXT)

Text S3 Gamma training set
(0.19 MB TXT)

Text S4 Lenti training set
(0.02 MB TXT)

Text S5 General training set
(0.36 MB TXT)
